# Palmitic acid is a toll-like receptor 4 ligand that induces human dendritic cell secretion of IL-1β

**DOI:** 10.1371/journal.pone.0176793

**Published:** 2017-05-02

**Authors:** Dequina A. Nicholas, Kangling Zhang, Christopher Hung, Shane Glasgow, Aruni Wilson Aruni, Juli Unternaehrer, Kimberly J. Payne, William H. R. Langridge, Marino De Leon

**Affiliations:** 1 Center for Health Disparities and Molecular Medicine, Loma Linda University School of Medicine, Loma Linda, California, United States of America; 2 Division of Biochemistry, Department of Basic Sciences, Loma Linda University School Medicine, Loma Linda, California, United States of America; 3 Department of Pharmacology and Toxicology, University of Texas Medical Branch, Galveston, Texas, United States of America; 4 Department of Basic Sciences, Division of Microbiology and Molecular Genetics, Loma Linda University School Medicine, Loma Linda, California, United States of America; 5 Department of Anatomy and Physiology, Loma Linda University School Medicine, Loma Linda, California, United States of America; 6 Department of Basic Sciences, Division of Physiology, Loma Linda University School Medicine, Loma Linda, California, United States of America; Universidade de Sao Paulo Instituto de Quimica, BRAZIL

## Abstract

Palmitic acid (PA) and other saturated fatty acids are known to stimulate pro-inflammatory responses in human immune cells via Toll-like receptor 4 (TLR4). However, the molecular mechanism responsible for fatty acid stimulation of TLR4 remains unknown. Here, we demonstrate that PA functions as a ligand for TLR4 on human monocyte derived dendritic cells (MoDCs). Hydrophobicity protein modeling indicated PA can associate with the hydrophobic binding pocket of TLR4 adaptor protein MD-2. Isothermal titration calorimetry quantified heat absorption that occurred during PA titration into TLR4/MD2, indicating that PA binds to TLR4/MD2. Treatment of human MoDCs with PA resulted in endocytosis of TLR4, further supporting the function of PA as a TLR4 agonist. In addition, PA stimulated DC maturation and activation based on the upregulation of DC costimulatory factors CD86 and CD83. Further experiments showed that PA induced TLR4 dependent secretion of the pro-inflammatory cytokine IL-1β. Lastly, our experimental data show that PA stimulation of NF-κB canonical pathway activation is regulated by TLR4 signaling and that reactive oxygen species may be important in upregulating this pro-inflammatory response. Our experiments demonstrate for the first time that PA activation of TLR4 occurs in response to direct molecular interactions between PA and MD-2. In summary, our findings suggest a likely molecular mechanism for PA induction of pro-inflammatory immune responses in human dendritic cells expressing TLR4.

## Introduction

Fatty acids (FAs) are potent regulators of immune function. For example, omega 3/6 polyunsaturated FAs are anti-inflammatory while saturated FAs stimulate inflammation [1]. Although lipids influence inflammation, mechanisms defining how FAs modulate immune cells remain unknown. Current theories include production of FA metabolites, a role for fatty acid binding proteins (FABPs), alteration of cell membrane composition, FA peroxidation, and more recently, receptor responses [2–4]. Although progress has been made in understanding FA-induced inflammation, further research regarding the mechanism of FA effects on immune cells remains crucial.

Palmitic acid (PA), a long chain saturated FA prevalent in the Western diet, may activate innate immune cells through TLR4. For example, PA activation of microglia is inhibited by TLR4 neutralizing antibodies, and dietary FAs were shown to activate TLR4 signaling in macrophages and to increase TLR4 dimerization [5–8]. Further, Lee et al. showed that saturated FAs did not activate NF-κB in TLR4 negative macrophage cell lines [9]. Although there is strong evidence that PA regulates immune cells through TLR4, no studies have yet demonstrated molecular interactions between FAs and TLR4.

Evidence suggests FAs may also be important contributors to the production of IL-1β, a pro-inflammatory cytokine regulated by TLR4 [10]. IL-1β is produced as a pro-protein in response to NF-κB activation through pathogen recognition receptor (PRR) signaling. PRR signaling is initiated through ligation from a pathogen associated molecular pattern (PAMP). However, this first signal which results in translation of pro-IL-1β is insufficient for secretion of the active cytokine. A second signal from TRIF/TRAM dependent TLR signaling, reactive oxygen species (ROS), or a damage associated molecular pattern (DAMP) such as uric acid, ATP, or DNA, is required for induction of pro-IL-1β cleavage prior to secretion. This second signal induces formation of an inflammasome, a multimeric protein complex that typically consists of a sensor molecule, an adaptor protein (ASC), and caspase [11]. NLRC4 (NOD-, LRR- and CARD-containing 4) is an example of a NOD-like receptor (NLR) that acts as a sensor molecule [12]. When pro-caspase-1 is recruited to the ASC via homotypic binding of CARDs (caspase activation and recruitment domains) [13], autolytic cleavage occurs, producing active caspase-1. Caspase-1 which then cleaves pro-IL-1β to form the active cytokine [11].

Because DCs express TLR4, they have the ability to secrete IL-1β, and play an important role in both innate and adaptive immune responses. PA has the potential to exert broad immune effects through activation of DCs. Dendritic cells are the dominant antigen presenting cells in the body and are responsible for conveying innate immune signals to lymphocytes; and therefore, it is of major importance to understand how they process various classes of immunogenic stimuli such as FAs. In innate immunity, the DC response to inflammation involves the secretion of TNF-α and nitric oxide to aid in the clearing of pathogens among other functions [14, 15]. In adaptive immunity, DC-secreted factors affect IgA antibody production by B cells [16]. Most importantly, DCs prime naïve T cells, stimulate Th1 and Th2 responses, cross present antigen (Ag) to CD8^+^ T cells, and regulate T cell differentiation [17, 18].

The ability of a DC to interact with T cells depends on its maturation (activation) state. Immature DCs are highly endocytic and express relatively low levels of T cell co-stimulatory markers and MHC [19, 20]. In contrast, mature DCs become mobile and express proteins important for T cell activation and have reduced antigen uptake, accompanied with efficient Ag presentation [21]. Phenotypically, high levels of CD83 expression is the best measurement of DC maturity [22]. DC activation is a process distinct from maturation. Activated DCs can be distinguished from resting mature DCs by expression of higher levels of MHC and CD86 and CD80 co-stimulatory molecules and by secretion of pro- or anti-inflammatory cytokines [23].

The goal of this study was to determine if PA functions as an agonist for TLR4 and to characterize PA-induced DC maturation and activation. We hypothesized that PA ligation of TLR4/MD-2 causes DCs to adopt a pro-inflammatory phenotype. Our findings show that PA binds TLR4 via the adaptor protein MD2 and that the TLR4 signal induced by PA stimulates DC activation and maturation accompanied by a robust IL-1β response. Thus, PA may function as a dietary immune modifier (DIM) resulting in the induction of chronic inflammation, such as that seen in type 2 diabetes.

## Materials and methods

### Fatty acid-MD-2 docking studies

Single ligand (fatty acid) to protein (MD-2 PDB Id#2e56) docking was performed using the Swissdock server [24]. Docking of multiple molecules of palmitic acid to MD-2 was performed using the Zdock server [25]. Docking Figures were generated with the UCSF Chimera package. Chimera was developed by the Resource for Biocomputing, Visualization, and Informatics at the University of California, San Francisco (supported by NIGMS P41-GM103311) [26].

### Monocyte isolation and MoDC culture

Human peripheral blood from healthy adult donors (age 18+) was provided by the Lifestream blood bank, San Bernardino, CA, according to Loma Linda University IRB requirements. The Loma Linda University IRB approved the use of blood from Lifestream blood bank for this study. All donors provided written informed consent collected and maintained by Lifestream blood bank, San Bernardino [27]. Monocytes were isolated using anti-CD14 magnetic beads and standard protocol (Miltenyi Biotech). The CD14^+^ (>97% purity) monocytes were cultured for 6 days in RPMI 1640 media supplemented with 10% FBS, 50 ng/mL GM-CSF, and 10 ng/mL IL-4 at a density of 1x10^6^ cell per mL of media, and used as a source of monocyte-derived dendritic cells (MoDCs).

### Preparation of palmitic acid (PA)

The PA (Sigma, St. Louis, MI) was dissolved in 100% EtOH and diluted in a range from 300 to 50 μM in a 2:1 molar ratio with endotoxin low FA-free BSA (Gemini Bio-products, shown not to induce PRR activity [28]) in media with charcoal stripped FBS (final concentration of EtOH is 0.1% v/v) [29]. The endotoxin low BSA contains less than 0.5 EU per mg of protein. In addition, to control for the possibility of minute levels of endotoxin, endotoxin low BSA was used in all experimental groups. Due to literature reports of BSA preparations possessing PRR agonist activity [28, 30], we performed control experiments to rule out endotoxin contamination of BSA as the source of palmitic acid’s effects. Via limulus assay, we found that our BSA preparations contain negligible amounts of endotoxin and did not elicit TLR4 associated cytokine secretion (S1A and S1B Fig). We found that using sodium palmitate instead of BSA-solubilized palmitic acid elicited a cytokine response from MoDCs, though on a smaller scale (S1C Fig)[31]. As was previously noted, sodium FAs induced robust responses from suspension cells such as THP-1 cells. However, PA solubilized with BSA is necessary for maximal responses from adherent cells [28, 31]. Finally, we showed that PA solubilized with BSA induces a cytokine response in the presence of 50ug/mL of the LPS sequestering agent polymyxin B sulfate (Sigma-Aldrich), a result previously achieved [1].

### Reagents

The reactive oxygen species (ROS) scavenger MCI-186 (Biomol Research Laboratories) was dissolved in DMSO and diluted in a range from 1mM to 50μM. The MoDCs were pre-treated with MCI-186 for 1hr. The TLR4 inhibitor CLI-095 (Invivogen, San Diego, CA) was used according to the manufacturer’s protocol. CLI-095 is a selective TLR4 inhibitor shown not to inhibit TLR2, 3, and 9 signaling [32]. Lipid IVa (Pepta Nova, Sandhausen, Germany), a lipid A antagonist which binds MD-2, was dissolved in DMSO and used at a final concentration of 1μg/mL. The caspase-1 inhibitor peptide (ICE Inhibitor V, Z-Asp-[(2,6-dichlorobenzoyl)oxy]methane, Z-D-CH2-DCB (Calbiochem,Billerica, MA) was used at 100μM final concentration. Control DC samples contained equimolar amounts of BSA, EtOH, or DMSO as used in experimental groups.

### Antibodies and flow cytometry

Antibodies and other reagents used were fixable viability dye e-flour 450, Annexin V FITC (ebioscience); anti-human CD86-FITC, CD80-FITC, Propidium Iodide (PI) (BD Pharmingen, San Jose, CA); anti-human CD11c PE, HLA-DR PerCP, CD80-PE (Becton Dickinson, San Jose, CA); CD282-FITC, CD284-PE, Donkey anti-rabbit-FITC, CD86-PE, CD83-APC, CD11c-PE-Cy7, CD83-FITC, CD14-APC, and 7 aminoactinomycin D (7AAD (Biolegend, San Diego, CA). Data acquisition was on a Miltenyi MacsQuant flow cytometer (Miltenyi Biotech, San Diego, CA) on a log scale and analysis was performed with FlowJo 7.6.5 software (Tree Star, Ashland, OR).

### Flow cytometry gating strategy

MoDCs were gated by FSC-A/SSC-A. Doublet discrimination was performed using FSC-H vs FSC-W and SSC-H vs SSC-W. Dendritic cells (CD11c^hi^cells >94%) all expressed increased HLA-DR as compared to monocytes (S2A–S2C Fig).

### PCR

MoDC mRNA was isolated using Stat-60 (Tel-Test Inc.) according to the manufacturer’s protocol. SydQuanti First Strand cDNA Synthesis Kit (SydLabs Inc., Natick, MA) was used for reverse transcription. qRT-PCR was performed with iQ SYBR Green Super Mix (Bio-rad, Irvine, CA) on a Bio-rad CFX96 Real-time system C1000^™^ thermal cycler. Primers were designed using NCBI Primer Blast.

### Cytometric bead array

Media from MoDC cultures following PA treatment was analyzed for cytokine concentration with human IL-12p40, IL-12/23p40, and IFN-α, IFN-γ, IL-6, TNF, and IL-1β flex sets as described by the manufacturer (BD Biosciences) on a MacsQuant flow cytometer. Data analysis was performed with FCAP array v3 software.

### Recombinant TLR4/MD2

Recombinant TLR4/MD2 (S2D Fig) was isolated from stably transfected HeLa cells according to published protocol [33]. An expression plasmid (vector pD1119 cloned by DNA 2.0, Menlo Park, CA) encoding puromycin resistance, and the TLR4 ectodomain (Accession # O00206; Glu24—Lys631 + 10-His Tag) and MD2 (Accession # Q9Y6Y9; Glu17—Asn160 +10-His Tag) separated by the sequence for a viral 2A peptide [34] was used to produce lentivirus. Transduced HeLa cells were expanded in DMEM + 10% FBS and 1μM puromycin (Sigma-Aldrich).

### Isothermal titration calorimetry

Isothermal titration calorimetry (ITC) experiments were carried out in a low volume Nano ITC (TA instruments, New Castle, DE). Electrical and chemical calibration was done according to the manufacturer’s instructions [35]. 200uL of 13–30μM recombinant human TLR4/MD-2 protein dissolved in ITC buffer (Tris/HCl pH 8, 1mM ethanethiol, 900μM KOH) was loaded into the reaction cell. The syringe was loaded with 50μL 500–1000μM PA. Titrations were carried out using 22 injections of ~2.5μL of PA into TLR4/MD-2 at 400s intervals, at 25°C. The data was analyzed using NanoAnalyze v2.4.1 software (TA Instruments). Integrated area values from the isotherms produced was fitted to an independent binding model in the NanoAnalyze software.

### Fluorescence microscopy

MoDCs were cultured on 8-well chambered glass slides (ThermoScientific, Pittsburgh, PA). After 20 min treatment with PA or PA+CLI-095, the MoDCs were fixed and stained with primary anti-human phospho NF-κBp65 monoclonal antibodies overnight at 4°C (Cell signaling Technologies, Danvers, MA) and secondary donkey anti-rabbit FITC (Biolegend). Slides were sealed after addition of mounting media with DAPI and photos were taken at 40x magnification on a Keyence BZ-9000 fluorescent microscope.

### Western blot and Electrophoretic Mobility Shift Assay (EMSA)

Western blots were performed on 50μg of total protein lysates from MoDCs treated with PA for reported time points to detect caspase-1 (anti-caspase-1 Cell Signaling Technology). EMSA was performed according to published protocol [36]. Nuclear lysates of PA treated MoDCs were incubated with a ^32^P labeled DNA NF-κB consensus sequence, 5′-AGTTGAGGGGACTTTCCCAGGC-3′ for 30min at room temperature. The protein/nucleotide mixtures were subjected to polyacrylamide gel electrophoresis as previously described [36]. The gels were dried and exposed on Fuji medical X-ray film. The band density correlates with the relative amounts of labeled oligonucleotide bound to NF-κB.

### Measurement of MoDC intracellular reactive oxygen species (ROS)

To measure ROS, MoDCs (2x10^6 cells/well in 6-well plates), both PA treated and untreated, were incubated with a final concentration of 25 μM 2’,7’-dicholorfluorescein diacetate (H2DCF-DA) (Invitrogen) for 20 min at 37°C. The negative control sample was treated with 1mM MCI-186 (Biomol Research Laboratories, Plymouth Meeting) for 24 hrs. The positive control samples were incubated with a final concentration of 10 μM H_2_0_2_ for 10min prior to the addition of H2DCF-DA. The H2DCF-DA readily diffuses into the cells where it is hydrolyzed to the polar non-fluorescent molecule 2’,7’-dicholorfluorescein (H2DCF). The DCFH is readily oxidized by ROS into the fluorescent molecule 2’,7’-dicholorfluorescein (DCF) within the cell [37]. The MoDCs were harvested and analyzed via flow cytometry. DCF fluorescence is indicative of the presence of oxidants produced from metal ion-, and peroxidase- catalyzed cellular reactions as well as carbon dioxide catalyzed decomposition of peroxynitrite. DCFH is not oxidized by superoxide, hydrogen peroxide, or nitric oxide, molecules most relevant to cellular process [38]. Therefore, the specific moiety PF6-AM was utilized.

PF6-AM was used to specifically measure H_2_O_2_. MoDCs, 2x10^6^, were cultured in 3cm^2^ plates. The MoDCs were then incubated in PBS with final concentration 5μM PF6-AM for 30 min at 37°C [39]. The MoDCs incubated with the PF6-AM were visualized on a Zeiss fluorescent microscope. The PBS with excess PF6-AM was removed and a baseline photo of fluorescence was taken. A photo was taken every 0.5s for 20s using SPOT ADVANCED software (Sterling Heights, MI). Media containing treatments was added and photos were initiated simultaneously. Fluorescence was quantified using Image J and normalized to baseline fluorescence to quantify H_2_O_2_ responses in MoDCs.

### PA-treated MoDC sample preparation and mass spectrometry analysis

The procedures used for proteomics analysis of protein analysis were adapted from our laboratory and others’ previously published protocols [27, 40–42]. The cell pellet was lysed in Radioimmunoprecipitation assay (RIPA) lysis buffer (Santa Cruz Biotechnology, CA) containing 1% Nonidet P40, PMSF (0.2mM), and protease inhibitor cocktail (Roche, one tablet per 10 mL). The protein mixture was centrifuged (>20,000 x g, 4°C) and the supernatant transferred into a clean tube. The protein concentration in the supernatant was determined by bicinchoninic acid assay (BCA assay). Approximately 60 μg of each protein sample was resuspended in 25mM triethylammonium bicarbonate buffer, pH 7.8. The protein solution was reduced by the addition of 10 mM TCEP (tris(2-carboxyethyl)phosphine) and incubation at 50°C for 30 min, followed by carboxymethylation with 25 mM iodoacetamide in the dark for 1 hr. The proteins were precipitated by the addition of 4 volumes of -200°C precooled acetone and stored at -200°C overnight. The protein was pelleted by centrifugation at 20,000 x g for 10 minutes and the supernatant was discarded. The protein was washed by 0.5 mL -200°C pre-cooled acetone and dried in chemical hood overnight after removal of acetone. The protein pellet was dissolved in 25 mM triethylammonium bicarbonate buffer and digested with trypsin at a protein / trypsin enzyme ratio of 25:1 (by mass) for 10 hours at 37°C. The TMT (Tandem Mass Tag) isobaric Mass Tagging Kit (Thermo-Fisher Scientific) was used to label the peptides by following the manufacturer’s recommendations. Specifically, for this experiment, monocytes were labeled by two TMT isotopes TMT-126 and TMT-127, MoDCs were labeled by two TMT-6 isotopes, TMT-128 and TMT-129, and MoDC + PA cells were labeled by two TMT-6 isotopes, TMT-130 and TMT-131. All the labeled samples were mixed together. After de-saltation with a spin-column with Hypercarb packing material (Thermo-Fisher Scientific), the eluted peptides with 60% acetonitrile were SpeedVac dried, re-dissolved in 1% formic acid, and then subjected to LC-MS/MS analysis. Quantitation was carried out on the Thermo QExactive mass spectrometer. Peptides were separated by online reverse phase liquid chromatography (RPLC) using home-packed C18 capillary columns (15 cm long, 75 μm i.d., 3-μm particle size) with a 250-min gradient (solvent A, 0.1% FA in water; solvent B, 0.1% FA in ACN) from 5–30% solvent B. Approximately 2 μg of peptide sample was injected. The Orbitrap mass analyzer was set to acquire data at 35,000 resolution (FWHM) for the parent full-scan mass spectrum followed by data-dependent high collision-energy dissociation (HCD) MS/MS spectra for the top 15 most abundant ions acquired at 7500 resolution. Four injections were performed.

### Data processing

Data were processed and searched using the Thermo Scientific Proteome Discoverer software suite 1.4 with Mascot search engine and the human data base (Swiss_Prot 2014.10). 10 ppm precursor ion mass tolerance and 0.1 Da fragment ion tolerance was applied for searching peptides. Peptides were filtered based on a false discovery rate cut-off of 1% (strict) and 5% (relaxed). Cysteine S-carbamidomethylation as fixed modification, methionine oxidation as variable modification, and up to two missing cleavages were considered during searching. At least one unique peptide with a score above 40 is considered as an authentic identification. The average ratios of two reporter ions (130+131)/(126+127) for DC+PC and (128+129)/(126+127) for DC were calculated for quantification. The protein expression ratios were used for IPA analysis and activation z-scores for up- (z>2) or down-(z<-2)[27]. A total of 1986 proteins were detected.

### Ingenuity pathway analysis of the PA-treated MoDC proteome

The Ingenuity Pathway Analysis program (IPA) is an intuitive web-based application for rapid and accurate analysis and interpretation of the biological meaning in genomic and proteomic data. Predicted protein-protein interaction networks and canonical pathways were generated from the mass spectrometer data analysis of dendritic cell proteins isolated before and after PA treatment by the Ingenuity Pathway Analysis program (IPA) Software (Ingenuity Systems, www.ingenuity.com, Qiagen, USA). Analysis of networks and pathways were made using log2 fold-changes and [-log (p-values)] between two-group comparisons. The ratios of significant protein expression levels were determined at r = 1.25. Muliple testing is corrected by using the Benjamini-Hochberg method.

### Analysis of Gene Expression Omnibus (GEO) data sets

The relative expression values for mRNA of CD1a, CD1b, and CD1c were extracted from the Geo Expression Omnibus data set (Data Set Record, GDS3963; Series, GSE26168), Pubmed Geo Profiles. As previously described, mRNA was isolated from primary adult blood and analyzed with tiling array by Karolina et.al [43]. The CD1 data from these experiments were graphed and analyzed using GraphPad Prism 6.00 software (GraphPad, San Diego, CA).

### Statistical analysis

One or two way-ANOVA was performed with GraphPad Prism 6.00 software (GraphPad, San Diego, CA), and values at p <0.05 were deemed significant (** = p<0.01, *** = p<0.001, **** = p<0.0001).

## Results

### PA induces MoDC maturation and secretion of IL-1β

**Fig 1:**
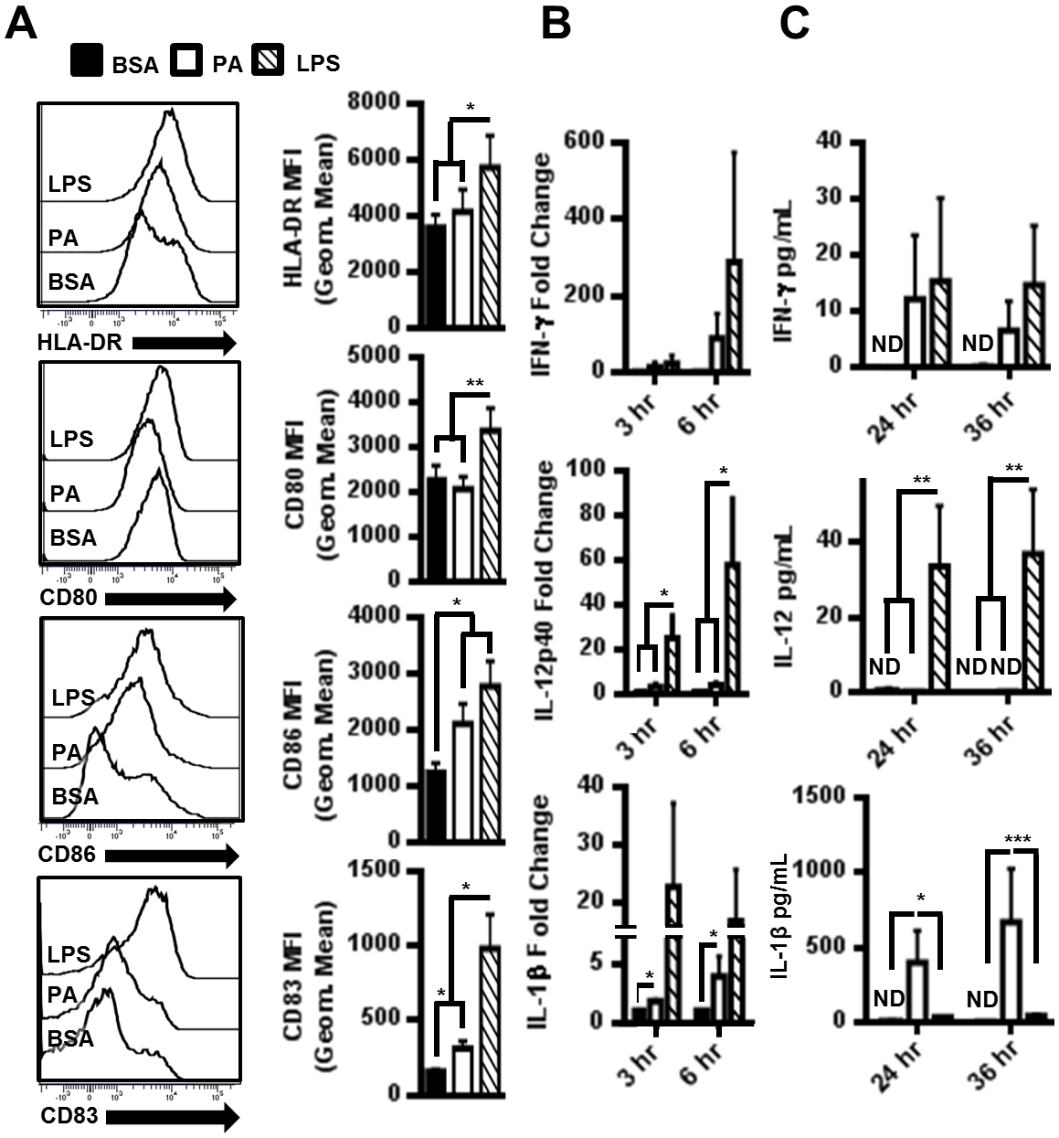
PA induced DC maturation and secretion of IL-1β. (A) PA upregulated DC activation and maturation markers. MoDCs were treated with 150μM PA for 12hrs. Representative histograms from 1 of 8 individual patient samples is presented, and the average geometric MFI for the indicated marker on CD11c^hi^ cells (MoDCs) from all 8 samples is graphed. (B) mRNA fold change in cytokines of MoDCs treated with 300μM PA (N = 8). (C) Quantification of cytokines secreted from MoDCs treated with 300μM PA (N = 4). ND = Not detectable.

Several studies have indicated the ability of free fatty acids to modulate DC differentiation and responses to LPS [9, 44–46]. However, the direct effect of PA on dendritic cell activation and maturation is unknown. Therefore, we assessed whether PA could induce MoDC activation and cytokine secretion. To determine if PA stimulates moDC maturation, we measured the upregulation of DC co-stimulatory factors. Naïve MoDCs were treated with PA and assessed for the surface expression of HLA-DR and CD80, CD86, and CD83 costimulatory factors by flow cytometry (Fig 1A). PA induced the expression of CD86 and CD83, indicating simultaneous induction of MoDC activation and maturation, while the polyunsaturated docosahexaenoic acid (DHA) had no effect (data not shown). We also examined the effects of PA on MoDC production of pro-inflammatory cytokines by qRT-PCR and cytometric bead array. PA increased IFN-γ secretion, although this trend was not significant (data not shown). PA had no effect on IL-12 production and failed to increase the secretion of IL-6, TNF-α, IL-8 or IL-10, but did increase the levels of IL-18 mRNA (data not shown). In contrast, PA induced robust expression of pro-IL-1β mRNA and the secretion of active IL-1β (Fig 1B and 1C). Interestingly, PA activated and LPS activated MoDCs differed in phenotype. LPS induced high levels of CD80, CD86, and CD83 while PA only moderately increased CD83 and CD86. Also, LPS induced secretion of a panel of pro-inflammatory cytokines with the exception of IL-1β, while PA selectively induced secretion of large amounts of IL-1β from MoDCs. These qualitative differences indicate that PA seems to elicit a more precise inflammatory response as opposed to LPS which has broader effects on cytokine production.

### PA is a TLR4/MD-2 ligand

**Fig 2:**
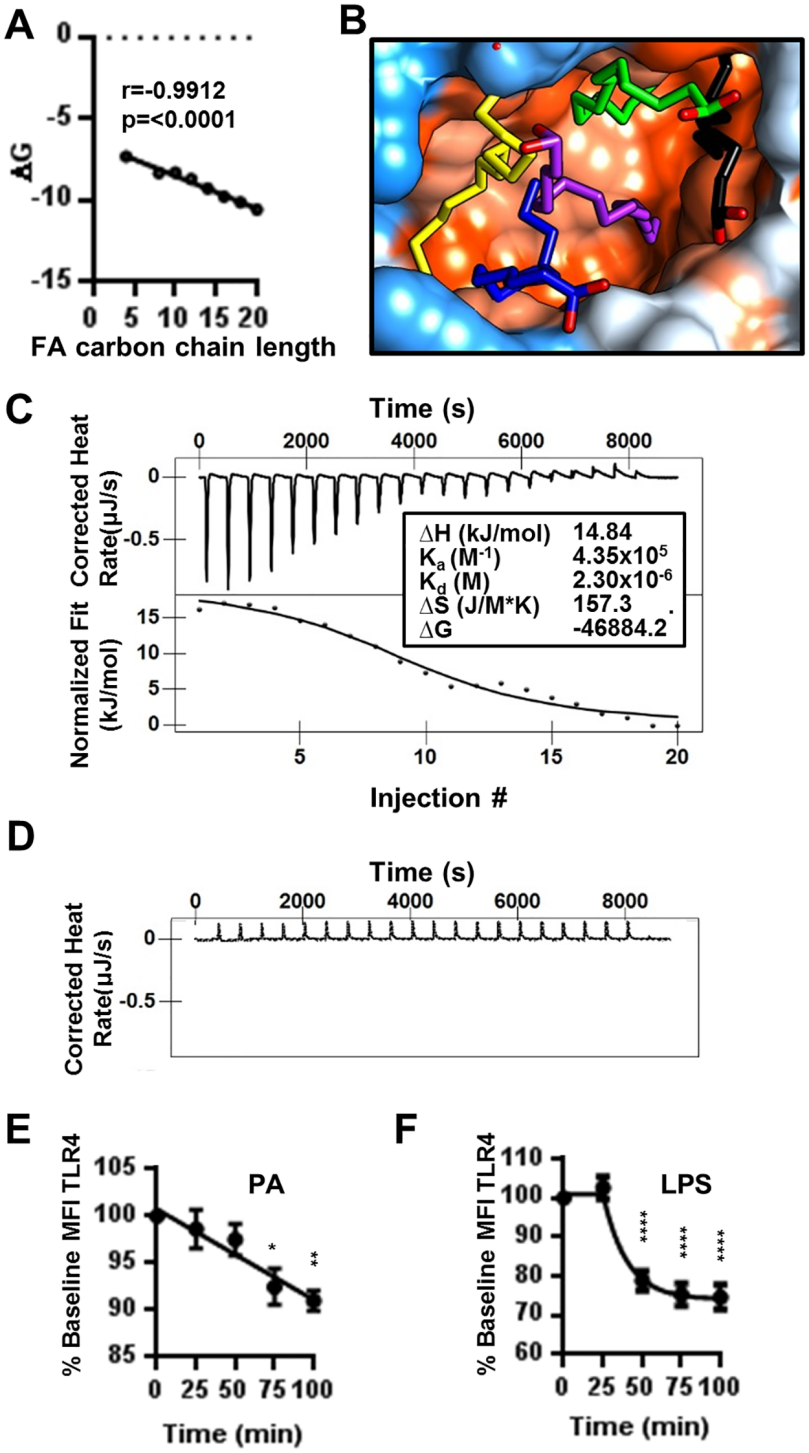
Palmitic acid is a ligand for TLR4/MD-2. (A) XY plot of fatty acid carbon chain length vs Gibbs free energy derived from docking FAs to MD-2 via the SwissDock server. (B) Molecular model of 5 PA docked in the hydrophobic binding pocking of MD-2. Orange color indicates hydrophobicity of MD-2 and blue indicates hydropholicity. Each PA molecule is in a solid color (yellow, black, blue, green, and purple) with oxygen atoms in red. (C) Isothermal titration calorimetry. Isotherms and fitted area curve of PA into TLR4/MD-2 titration. Insert contains thermodynamic parameters for PA and TLR4/MD-2 binding interaction. (D) Control isotherm of PA titrated into buffer. (E-F) PA induces the internalization of TLR4. MoDCs were treated with 300μM PA (E) or 10ng/mL LPS (F) for indicated time intervals. Cells were stained for flow cytometry. CD14^-^CD11c^hi^HLA-DR^hi^ cells were assessed for TLR4. Data is presented as the percentage of baseline Geometric MFI. N = 3. Graphs display the mean ± SEM. Statistical analysis was calculated based on One way ANOVA (** = p<0.01, *** = p<0.001, **** = p<0.0001).

Although it is undisputed that saturated FFA can induce inflammation by activation of TLRs, the mechanism remains unknown. We propose that saturated fatty acids (FAs) such as PA are direct TLR4/MD-2 ligands. Natural TLR4 agonists like Lipopolysaccaride (LPS) do not directly bind to the receptor. Rather, the association is mediated by the adaptor protein MD-2. The crystal structure of the TLR4 ectodomain, MD-2, and LPS show that five hydrophobic carbon chains of the lipid A portion of LPS bind within the hydrophobic pocket of MD-2 [47]. Due to structural similarity between the carbon chains of lipid A and FAs, we hypothesized that FAs may bind the hydrophobic pocket of MD-2. To test this hypothesis, we performed ligand-protein docking using the SwissDock server to develop a model of how saturated FAs bind to MD-2 in addition to the predicted change in Gibb’s free energy (ΔG) for the interaction [24]. The docking studies revealed that indeed saturated FAs have the potential to associate with MD-2 (S3A Fig). However, in this model, MD-2 is unable to discriminate between saturated and unsaturated FAs (data not shown). In addition, this theoretical approach resulted in a negative ΔG for all ligands, indicating that if these associations occurred, it would be spontaneous. As the length of the FA carbon chains increased, the ΔG became more negative (Fig 2A), indicating that increased ligand hydrophobicity facilitates association with the binding pocket of MD-2.

Although saturated FAs theoretically bind MD-2, it is unlikely that a single fatty acid could activate TLR/MD-2 upon ligation *in vivo*. Structure-function studies of TLR4/MD-2 and Lipid A indicate that LPS with fewer than 5 fatty acid chains in the binding pocket of MD-2 did not elicit biological activity from the receptor [47, 48]. Therefore, we performed further docking studies using the Z dock server to model the structure-function relationship between MD-2 and its natural ligands. In particular, we analyzed the association of MD-2 with multiple molecules of palmitic acid (PA), the most abundant FA in the human body. By mimicking the orientation of Lipid A carbon chains, we demonstrated the possibility for fitting 5 molecules of PA into the hydrophobic binding pocket of MD-2 as hypothesized (Fig 2B and S3B–S3D Fig).

To experimentally verify the docking of PA to MD-2, we employed isothermal titration calorimetry (ITC) to measure heat changes (ΔH) from the direct interaction of PA with TLR4/MD-2. As opposed to methods such as FRET or pull-down assays, ITC provides advantages such as not requiring labeling or immobilization in addition to the knowledge of all molecules involved in the interaction. Our results show that PA binds to TLR4/MD-2 with an affinity (K_a_) of 4.355x10^5^ 1/M (Fig 1C and 1D). The binding of PA to TLR4 is endothermic, exergonic, and spontaneous as evidenced by a positive ΔH, large positive ΔS, and negative ΔG respectively (Fig 2C). Because it has been previously shown that LPS induces TLR4 internalization in macrophages and MoDCs upon receptor ligation, we provided further indirect support of the interaction of PA with TLR4/MD-2 by assessment of MoDC internalization of TLR4 in response to PA [49]. PA was shown to significantly reduce surface TLR4 (Fig 2E) but to a lesser degree than LPS (Fig 2F).

### Palmitic acid exerts pro-inflammatory activity through TLR4

**Fig 3:**
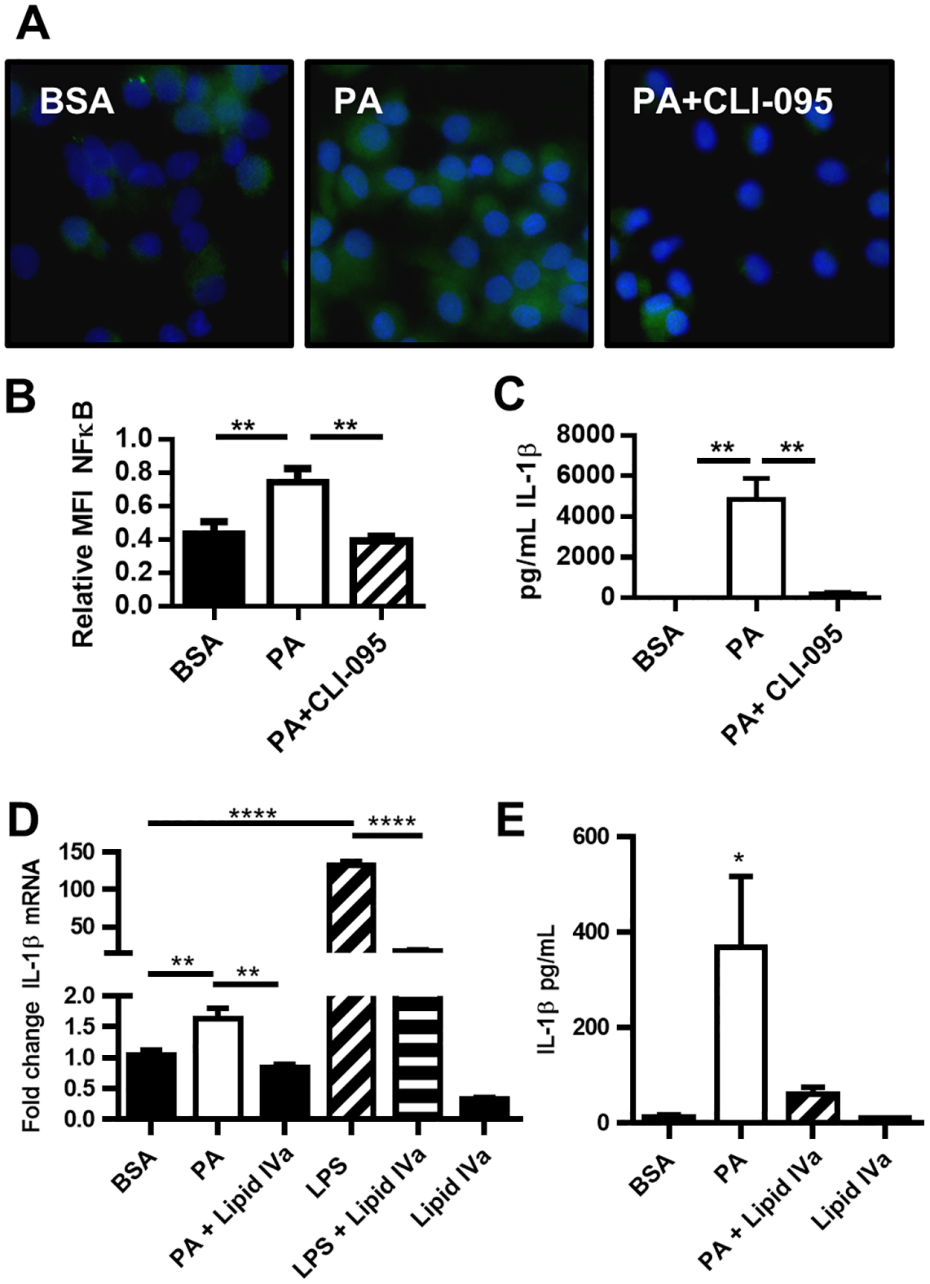
PA activation of NF-κB is TLR4 dependent. (A-B) PA-induced phosphorylation of NF-κBp65 is TLR4 dependent. MoDCs were treated with 300μM PA for 20 min and analyzed for phospho NF-κBp65 (green) by fluorescence microscopy. (B) Quantitation of MFI phospho NF-κBp65 relative to DAPI (blue). (C) MoDC secretion of IL-1β is dependent on TLR4 signaling. MoDCs were treated with 150μM PA for 36hrs +/- CLI-095. The supernatant was analyzed for the concentration of IL1-β. (D) MoDC production of IL-1β mRNAis dependent on TLR4 signaling. MoDCs were treated with 150μM PA for 6hrs +/- Lipid IVa. mRNA was isolated and RT-qPCR was performed. (E) MoDC secretion of IL-1β is dependent on TLR4 signaling. MoDCs were treated with 150μM PA for 36hrs +/- Lipid IVa. The supernatant was analyzed for the concentration of IL1-β. N = 3.

To further demonstrate the likelihood that PA is a direct TLR4/MD-2 ligand, we assayed the activation of NF-κB as a downstream effecter in TLR4-induced activation of MoDCs [50]. We determined that PA induces phosphorylation of the canonical NF-κB subunit p65 (Figs 3A, 3B and 6E). Activation of NF-κB is inhibited by blocking TLR4 with CLI-095 (Fig 3A and 3B). Next, we determined that blocking TLR4 with CLI-095 eliminates the IL-1β response to PA (Fig 3C). Therefore, ligation of PA to TLR4 is necessary for PA to induce IL-1β secretion from human MoDCs. In further evidence of PA acting as a TLR4 agonist through binding of MD-2, we tested the ability of lipid IVa, a synthetic lipid that occupies the MD-2 hydrophobic pocket [51] to block PA-induced IL-1β secretion. We found that lipid IVa does indeed block PA induction of IL-1β mRNA and protein (Fig 3D and 3E).

### Palmitic acid induces MoDC secretion of IL-1β through a classical caspase 1 mechanism

**Fig 4:**
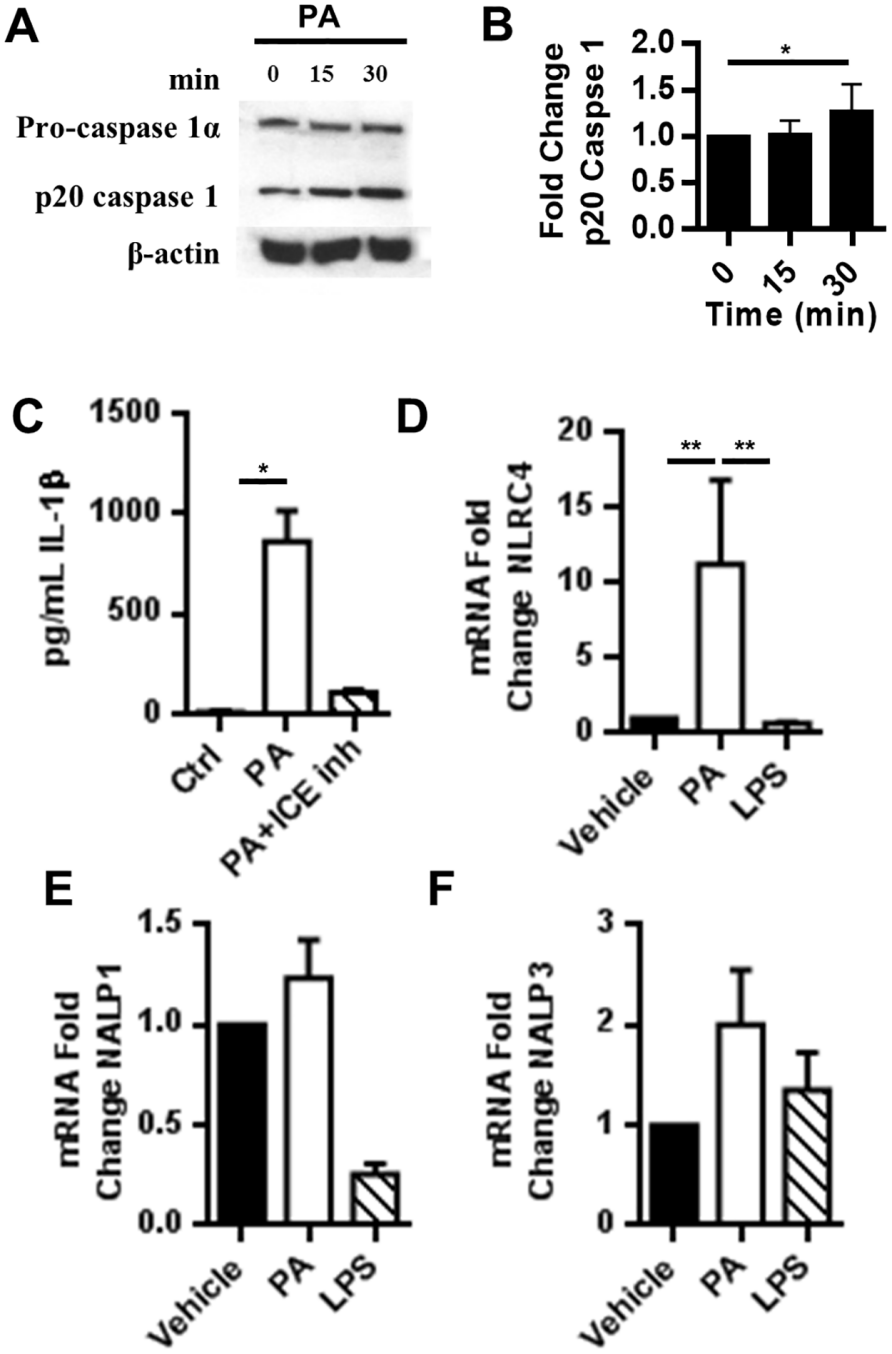
IL-1β is processed via caspase 1. (A-B) PA induces activation of caspase-1. MoDCs were treated with 300μM PA for the indicated times. The MoDC lysates were analyzed by western blot. (B) Bar graph is the average of 3 experiments and normalized to β-actin. (C) PA secretion of IL-1β is caspase-1 dependent. The supernatant from MoDCs treated with 300μM PA for 24hrs was analyzed by cytometric bead array. (D-F) PA induces inflammasome gene expression. MoDCs were incubated with 300μM PA for 6hrs and NLRC4, NALP1, and NALP3mRNA was analyzed by qRT-PCR.

Unlike the other cytokines induced by PA, IL-1β must be processed before secretion from the cell. Although, caspase-1 typically processes pro-IL-1β, caspase-8, also induced by TLR4, can cleave pro-IL-1β [52]. To determine whether PA-induced secretion of pro-IL-1β in MoDCs relies on caspase-1 cleavage, we assessed the ability of PA to induce activation of caspase-1 in MoDCs. Our results showed that PA increased the production of active p20 caspase-1 (Fig 4A and 4B). To evaluate whether caspase-1 activity is essential for IL-1β secretion, we assessed the secretion of IL-1β in the presence of a specific caspase-1 inhibitor (ICEinh). The inhibition of caspase-1 drastically reduced IL-1β secretion (Fig 4C). For caspase-1 to function, an active inflammasome must be formed. Thus, we assessed the mRNA expression of inflammasome genes in PA-treated MoDCs to determine which inflammasome is likely to associate with caspase-1 in response to PA. We found that PA specifically increases NLRC4 inflammasome mRNA approximately 10-fold compared to the untreated control without affecting NALP1 or NALP3 mRNA levels (Fig 4D–4F).

**Fig 5:**
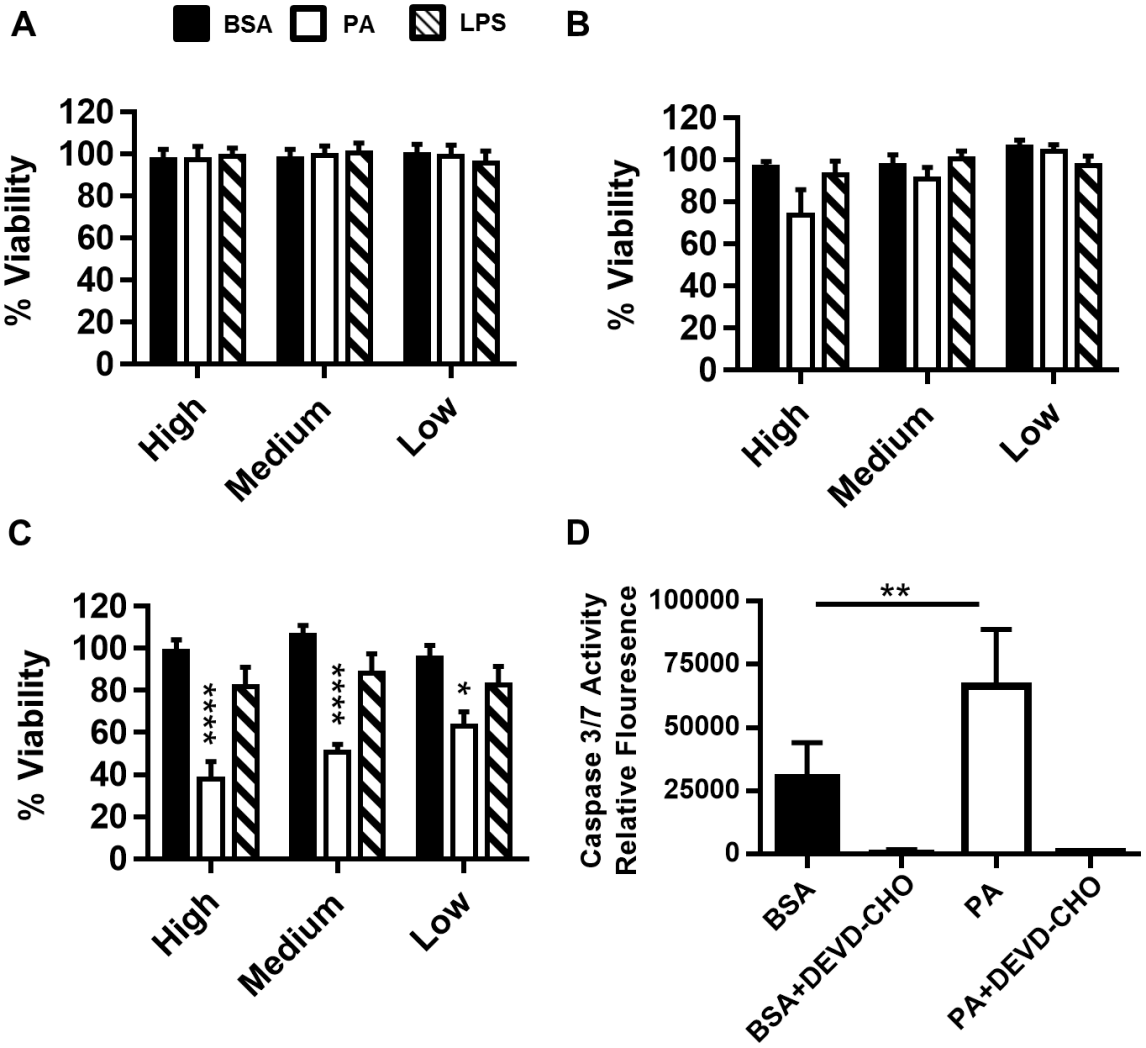
Dendritic cell viability following high (300μM), medium (150μM) and, low (75μM) dose PA treatment. MoDCs were inoculated with PA in a 2:1 molar ratio with BSA for (A) 12 hrs, (B) 24 hrs, or (C) 48 hrs. The cells were analyzed for loss of viability by flow cytometry using Annexin V and propidium iodide (PI) staining. LPS was used as a positive control to assess MoDC activation. The data is represented as the percent of total MoDCs negative for both Annexin V and PI normalized to the BSA negative control. (D) PA induces caspase 3/7 activity. MoDCs were treated with PA complexed 2:1 with BSA for 36 hrs. Caspase 3/7 activity was measured by the emitted fluorescence of cleaved DEVD-AMC. N = 3. Statistical analysis was calculated based on One way ANOVA (** = p<0.01, *** = p<0.001, **** = p<0.0001).

In addition to processing and maturing IL-1β, caspase-1 also triggers pyroptosis, a form of programmed necrosis that results in cell rupture and subsequent spilling of cellular contents to promote inflammation [53, 54]. It has been recently published that IL-1β release and pyroptosis are processes coupled by the protein gasdermin D [55, 56]. Because cleavage of gasermin D is required for IL-1β release and is sufficient to drive pyroptosis, we hypothesized that PA induced activation of caspase-1 would result in programmed cell death. By 48hrs, all concentrations of PA tested reduced cell viability (Fig 5A–5C). PA-induced cell death was associated with activation of caspase 3/7, implicated in both apoptosis and pyroptosis (Fig 5D).

### Palmitic acid activation of MoDCs is associated with ROS

**Fig 6:**
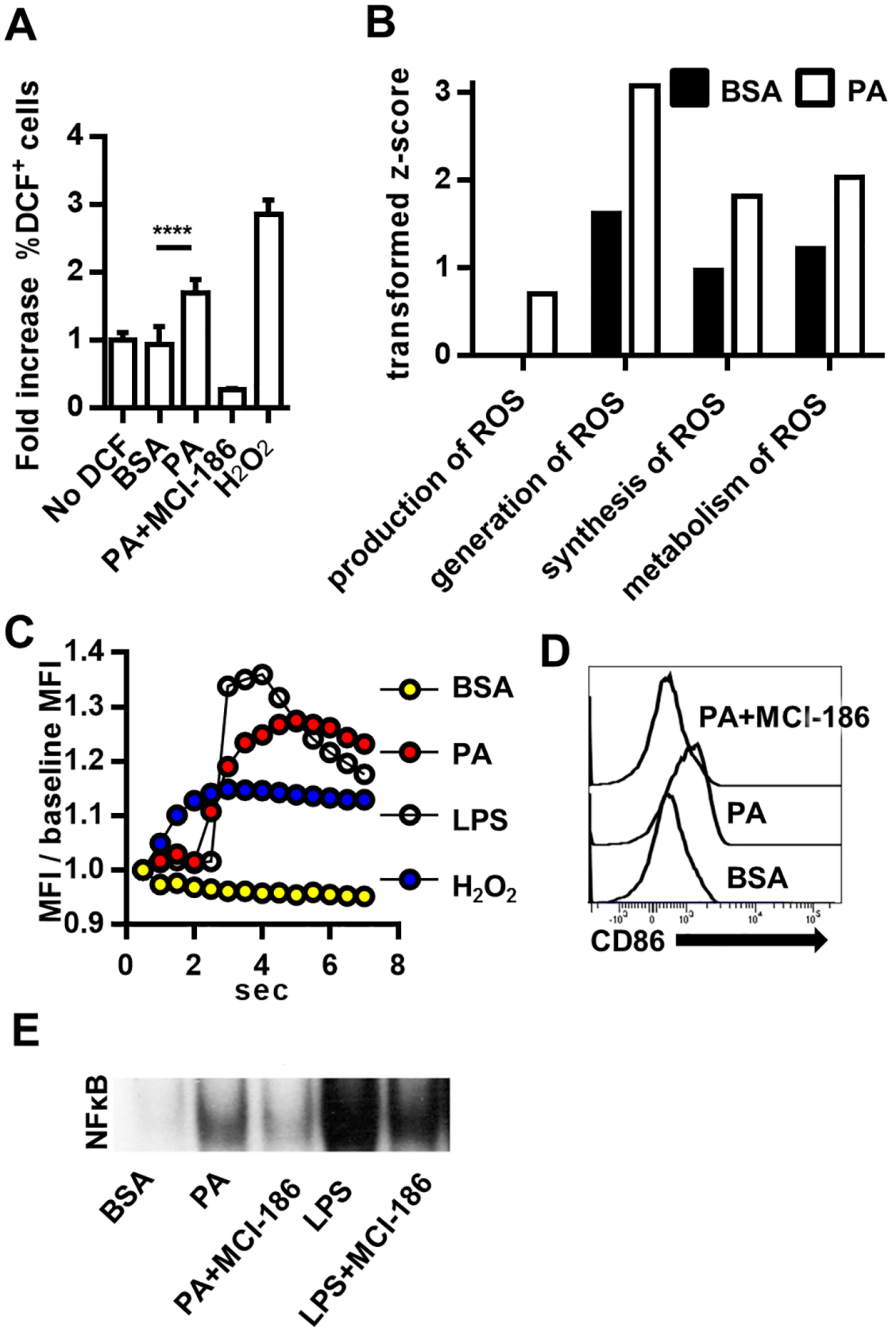
PA activation of MoDCs is regulated by ROS. (A) PA induces ROS in MoDCs. MoDCs were treated for 24hrs with PA. ROS was measured by DCF fluorescence and expressed as fold increase relative to MoDCs not incubated with DCF. The ROS scavenger MCI-186 was used as a negative control. N = 3. (B) Comparison of activation z-scores for cellular functions related to ROS production. After 24hr treatment, the DC proteome was assessed by mass spectrometry and protein changes analyzed using IPA. Transformed z-scores are displayed on the bar graph. The z-scores indicate the association of proteome changes with the indicated cellular functions in the MoDC after PA treatment. Non-transformed z-scores and p-values for association with cellular functions are in Supplement 4. (C) MoDCs were pre-incubated with the H_2_O_2_ probe PF6-AM. Upon addition of the indicated stimuli, PF6 fluorescence was detected by rapid-fire snap shots. The mean fluorescence intensity (MFI) of PF6 was normalized to baseline and plotted against time. (D) PA-induced MoDC activation is attenuated by MCI-186. MoDCs were treated with 300μM PA for 36 hrs. Cells were stained and analyzed by flow cytometry. MoDCs were gated by the phenotype (CD11c^hi^) and histograms for CD86 were plotted. Data is representative of three separate experiments. (E) PA-induced activation of NF-κB is ROS dependent. MoDCs were treated with 300μM PA+/- MCI-186 or LPS+/- MCI-186 for 3hrs and analyzed by EMSA.

Our laboratory and others have shown that cellular exposure to high levels of PA results in increased cellular ROS [57–59]. To confirm PA generation of ROS in MoDCs, we first directly measured ROS in PA treated MoDCs. As expected, PA increased the levels of ROS after 24hrs of treatment (Fig 6A). Ingenuity pathway analysis of proteome changes induced by PA in MoDCs after 24hrs supported this finding (Fig 6B). Because PA stimulates TLR4 and TLR4 has been shown to activate NADPH oxidase 4 (NOX4) [60], we were also interested in PA-generated H_2_O_2_ oxidative bursts that may contribute to regulation of TLR4 signaling in MoDCs [61]. H_2_O_2_ oxidative bursts would be observed at very early time points. Using fluorescence microscopy and the selective H_2_O_2_ probe PF6, we showed that PA, similar to LPS, induced H_2_O_2_ production in dendritic cells within seconds after PA addition (Fig 6C) [39].

Because PA induces H_2_O_2_ in MoDCs and because the inhibition of ROS with MCI-186 abrogated PA-induced TLR4 signaling, we hypothesized that PA-induced ROS from NOX4 acts as a TLR4 signaling regulator [9, 62, 63]. To characterize the role that PA generated ROS plays in PA induced DC activation, we co-treated MoDCs with PA and MCI-186 (a non-specific ROS scavenger). Our results showed that blocking ROS resulted in the inhibition of PA-upregulated MoDC CD86 (Fig 4D). Lastly, using EMSA, we assessed the effect of PA-induced ROS on PA-induced NF-κB activation. Blocking ROS with MCI-186 considerably reduced the levels of active NF-κB generated by MoDCs in response to PA (Fig 6E). These results suggest that PA-induced DC activation is partially dependent on ROS.

### Palmitic acid impairs MoDC antigen presentation

**Fig 7:**
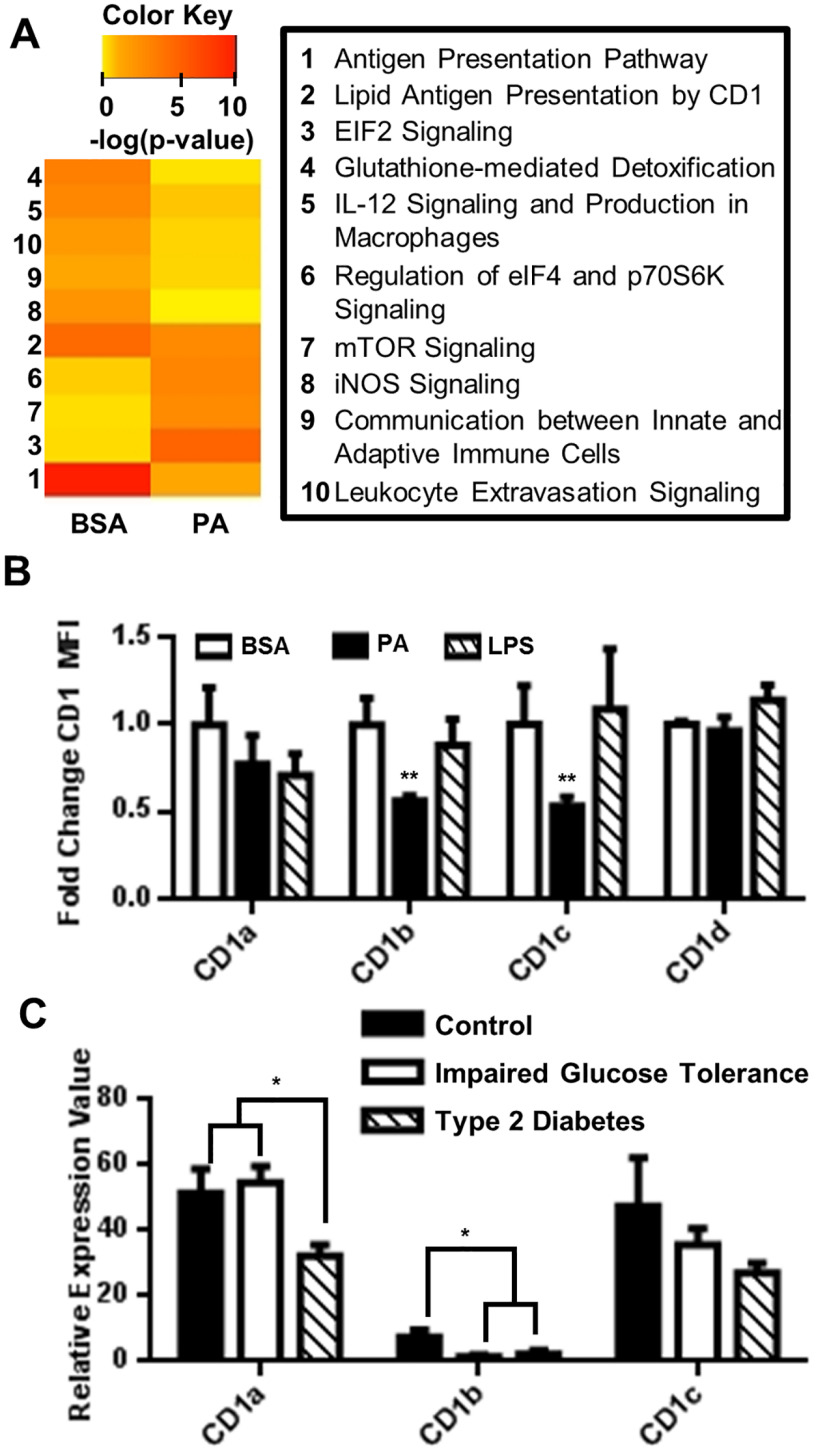
PA downregulates DC expression of CD1. (A) Heat map representing canonical pathways [-log(p-values)] identified in PA-treated MoDCs. Increasing intensity of red color indicates a higher probability that the indicated pathway is regulated by PA. (B) MoDCs were treated with 150μM PA complexed 2:1 with BSA for 24hrs. Following incubation, the cells were stained for surface receptors and analyzed by flow cytometry. The normalized MFI for the indicated markers on CD11c^hi^ MoDCs was plotted. N = 3. (C) CD1 downregulation is associated with type 2 diabetes. Analysis of public Geo Expression Omnibus data set (Data Set Record, GDS3963; Series, GSE26168) is presented. mRNA from primary adult blood was analyzed with tiling array by Karolina et.al [43]. (Controls, N = 8), (Impaired fasting glucose, N = 7), (Type 2 Diabetes, N = 9). Statistical analysis was performed using One way ANOVA (** = p<0.01, *** = p<0.001).

After demonstrating that PA-induces MoDC activation and secretion of IL-1β, we set out to further characterize the effects of PA on DC function. We used mass spectrometry to measure changes in the PA-treated DC proteome. The significance of detected protein changes was further assessed using ingenuity pathway analysis (IPA). The IPA program uses algorithms to identify signaling pathways most likely associated with proteomic changes caused by PA treatment. The significance of these associations is indicated by p-values, and with these p-values, we generated a heat map (Fig 7A). By comparing protein changes between PA-treated MoDCs and the control MoDCs (BSA treatment only), we found that PA treatment decreased the association of MoDCs with four important pathways: “Antigen Presentation (1),” “Lipid Antigen Presentation by CD1 (2),” “Leukocyte Extravasation Signaling (10),” and “Communication between Innate and Adaptive Immune Cells (9)” (Fig 6A). Paradoxically, IPA indicates that PA impairs classical DC function, while our *in vitro* studies demonstrate that PA induces a specific IL-β secreting phenotype, thus highlighting another qualitative difference between PA and LPS activation of MoDCs.

### Palmitic acid downregulates MoDC CD1 molecules

Antigen presentation is a crucial step in adaptive immune responses. Therefore, it is imperative to understand how saturated FAs may affect this process. Literature reports indicate that saturated FAs impair protein antigen presentation and that serum lipids regulate the CD1 surface molecules involved in lipid antigen presentation [64, 65]. Because our mass spectrometry data indicated a decreased association with “lipid antigen presentation by CD1,” we assessed changes in surface expression of CD1 molecules on MoDCs in response to PA treatment by flow cytometry. We found that CD1b and CD1c molecules were significantly down-regulated on the surface of MoDCs in response to PA (Fig 7B). Because diet associated inflammatory diseases such as type 2 diabetes can be characterized by high serum lipids such as PA, we hypothesized that metabolically unhealthy patients would have decreased CD1 expression. We tested this hypothesis by analyzing a public data set for CD1 molecule expression among metabolically healthy adults, pre-diabetic adults, and adults with type 2 diabetes and we found that CD1a and CD1b are significantly downregulated in the diseased state (Fig 7C). Although in this cohort, CD1 may be regulated by a variety of factors, this result suggests that saturated FA downregulation of CD1 is a potential mechanism of immunological dysfunction to investigate in the development of type 2 diabetes. Future studies should focus on demonstrating impaired antigen presentation in MoDCs treated with PA or in MoDCs derived from pre-type 2 diabetic patients and patients with type 2 diabetes.

## Discussion

Currently, the mechanisms underlying FA regulation of innate immunity are obscure. In 2006, Shi et al. demonstrated for the first time that dietary FAs activate TLR4 signaling in macrophages [5]. Additionally, the authors found that FA-induced inflammatory signaling was blocked in the absence of TLR4. While the study provided a link between nutrition and innate immunity, it did not show the mechanism of how FAs regulate the innate immune response. We now report for the first time, data demonstrating that PA can directly trigger innate immune responses by acting as a TLR4/MD-2 ligand (Figs 2 and 3).

The first step in PA modulation of innate immunity is binding to TLR4/MD-2. Our data show that PA binds TLR4 with a K_d_ of 2.3μM. The high affinity of PA for TLR4/MD-2 suggests that an increase in dietary PA may stimulate innate immune cells *in vivo*. PA’s association with TLR4/MD2 is three orders of magnitude weaker than that of LPS interaction with MD-2 (K_d_~ 3nM) [66]. However, the K_a_ of PA binding to TLR4/MD-2 is greater than that of PA binding to any of the three low affinity binding sites of human serum albumin, the natural carrier of FAs in the blood [67]. Under normal conditions, the low affinity binding sites are unoccupied. However, these sites become occupied under conditions of increased lipolysis and serum FAs, such as in obesity. Therefore, both serum PA and PA bound to low affinity sites could potentially bind to TLR4/MD-2 *in vivo*.

Palmitic acid was shown to be positively correlated with the incidence of type 2 diabetes development [68–70]. An emerging mechanism for PA contribution to the development of type 2 diabetes is the modulation of innate immune cell cytokine profiles [71–73]. In agreement with data generated from a murine model by Reynolds et al., we showed that PA activates human DCs, leading to IL-1β secretion (Fig 1C) [71]. The importance of IL-1β in the development of type 2 diabetes is supported by several *in vitro*, *in vivo*, and clinical studies [74–78]. Because PA induces secretion of IL-6 from macrophages and our data demonstrates PA induces secretion of IL-1β from human MoDCs, it is possible to suggest that PA may exacerbate the development of type 2 diabetes through stimulation of innate immune cells. IL-1β supports the development of Th17 cells [79, 80], and Th17 cells have recently been implicated as a predominant driver of inflammation in human T1D and T2D [81]. In mice, PA primes MoDCs to generate Th1/Th17 responses upon LPS stimulation [44]. This study found no direct effect of PA on human MoDCs, likely due to too short of a treatment time for PA, a weak TLR4 agonist as we have demonstrated here. Next steps would include directly testing whether PA activated DCs support Th17 differentiation *in vitro*.

PA induction of ROS may be an important mode of regulation for TLR4 signaling in human DCs. In support of this observation, our data show that an ROS inhibitor significantly prevents PA-induced NF-κB activation (Fig 4). The activation of NF-κB is an essential step in PA-induced DC secretion of IL-1β. Thus, PA ligation of TLR4/MD-2 and ROS generation are both required for PA to exert its maximum immune-stimulatory effects. Exposure of bone marrow-derived DCs to saturated FAs were shown to increase NLRP3 inflammasome activation [71]. MoDCs from mice on a high fat diet are similarly primed to secrete high levels of IL-1β likely due to chronic exposure to elevated FAs *in vivo* [71]. Our data indicate that the NLRC4 inflammasome, rather than NLRP3, maybe active in processing pro-IL-1β following PA activation of DCs (Fig 5). This difference may be due to our use of PA rather than a mixture of saturated FAs or may be cell type dependent. In defense of our observation, Luo et al. demonstrated that PA specifically activates NLRC4 in HepG2 cells [82]. Additionally, this distinct ability of PA to activate inflammasomes and induce IL-1β secretion has been demonstrated in murine hepatocytes [83].

This manuscript begins to address the mechanism by which PA modulates innate immunity. However, many gaps in the literature, such as the role of FABPs in PA, remain unexplored. FABP4-deficient macrophages and dendritic cells have an impaired ability to produce cytokines upon stimulation [84, 85], but the mechanism is unknown. Another gap in the literature is the mechanism by which LPS activates MoDCs. Our data demonstrates that treating MoDCs with LPS results in internalization of TLR4 (Fig 2). However, human MoDCs do not express CD14, the co-receptor demonstrated to be necessary for TLR4 internalization in mouse macrophages and DCs [49]. A recent report of ligands that induce CD14 independent internalization of TLR4 [86] and the induction of CD14 upon activation [49], provides a basis for answering this important question.

Overall, our studies suggest that PA is a metabolic ligand for TLR4/MD-2 that stimulates production of IL-1β in human DCs through an inflammasome dependent mechanism (S4 Fig). Future studies utilizing FRET and pull down assays in addition to investigating known co-receptors for TLR4 such as CD14 will be important in developing binding models of FAs to TLRs. The potential role of PA as a dietary immune modulator (DIM) in diseases such as type 2 diabetes underscores the importance of researching lipid effects on immune cell responses. The emergence of mechanistic data on DIMs such as FAs has the potential to provide novel molecular targets for therapy in a host of inflammatory diseases influenced by diet.

## Supporting information

S1 Fig The effects of palmitate on MoDC activation are not due to LPS contamination. (A) Limulus assay of RPMI 1640 media with 10% FBS (Ctrl), Ctrl + 300μM PA, 150μM BSA, 150μM BSA + 300μM PA, and 10ng/mL LPS. One way ANOVA (** = p<0.01, *** = p<0.001, **** = p<0.0001). (B) MoDCs were treated with 150μM PA for 36hrs +/- Lipid IVa. The supernatant was analyzed for the concentration of IL-12. N = 3. One way ANOVA (** = p<0.01, *** = p<0.001, **** = p<0.0001). (C) MoDCs were treated with 150μM sodium palmitate (NaPA) +/- Polymyxin B or Lipid IVa for 36hrs. The supernatant was analyzed for the concentration of IL-1β. N = 4. (D) MoDCs were treated with 150μM palmitate solubilized with BSA (BSA-PA) +/- Polymyxin B or Lipid IVa for 36hrs. The supernatant was analyzed for the concentration of IL-1β. N = 4. ND = Not detectable. Two-way ANOVA (** = p<0.01, *** = p<0.001, **** = p<0.0001) compared to control unless indicated. All cytokines were detected using the Th17 multiplex kit (Millipore) and measured on the Bio-Plex^®^ 3D System with Luminex xMap Technology (Bio-Rad), (more sensitive and with a larger dynamic range than the cytometric bead array system).
(TIF)

S2 Fig Basic MoDC gating strategy. (A) MoDCs were gated first by FSC-A and SSC-A. Doublet discrimination was performed using FSC-H vs FSC-W and SSC-H vs SSC-W. Cells with the phenotype CD11c^hi^ were gated as MoDCs (>94%). (B) DCs express higher levels of CD11c than their precursor monocytes. (C) DCs express higher levels of HLA-DR than their precursor monocytes. (D) Western blot and Coomasie stain of SDS-PAGE of isolated rTLR4 and rMD-2 from Hela cells.
(TIF)

S3 Fig PA binding model. (A) SwissDock model of PA bound to the hydrophobic pocket of MD-2. Orange color indicates hydrophobicity of the protein and blue indicates hydropholicity. (B) Five palmitic acid molecules oriented based on the structure of LPS bound to MD-2. Each PA molecule is in a solid color (yellow, black, blue, green, and purple) with oxygen atoms in red. (C) Hydrophobicity molecular model of five palmitic acid molecules from (B) bound within the hydrophobic pocket of MD-2. (D) Model of five palmitic acid molecules bound to MD-2 (translucent).
(TIF)

S4 Fig Mechanism of PA-induced DC secretion of IL-1β. (1) PA binds TLR4/MD-2 and induces signal transduction resulting in NF-κB activation. (2) The canonical NF-κB signaling pathway induces transcription of the pro-IL-1-β gene which results in translation and protein expression. (3–4) PA-induced TLR4 signal transduction also results in activation of caspase-1, a process which occurs via inflammasome assembly. (5) Caspase-1 cleaves pro-IL-1-β into active IL-1-β. (6) With the secretory peptide signal exposed after cleavage, IL-1β is secreted from the cell. ROS has an undefined role in regulating this mechanism.
(TIF)

S5 Fig PA-induced activation of NF-κB is ROS dependent. An uncropped image of Fig 6E. MoDCs were treated with 300μM PA+/- MCI-186 or LPS+/- MCI-186 for 3hrs and analyzed by EMSA.
(TIF)
