# Supplementary figures and images for: Palmitic acid is a toll-like receptor 4 ligand that induces human dendritic cell secretion of IL-1β
Source: PLoS One. 2017 May 2;12(5):e0176793. doi: 10.1371/journal.pone.0176793 (PMC5413048; doi:10.1371/journal.pone.0176793)

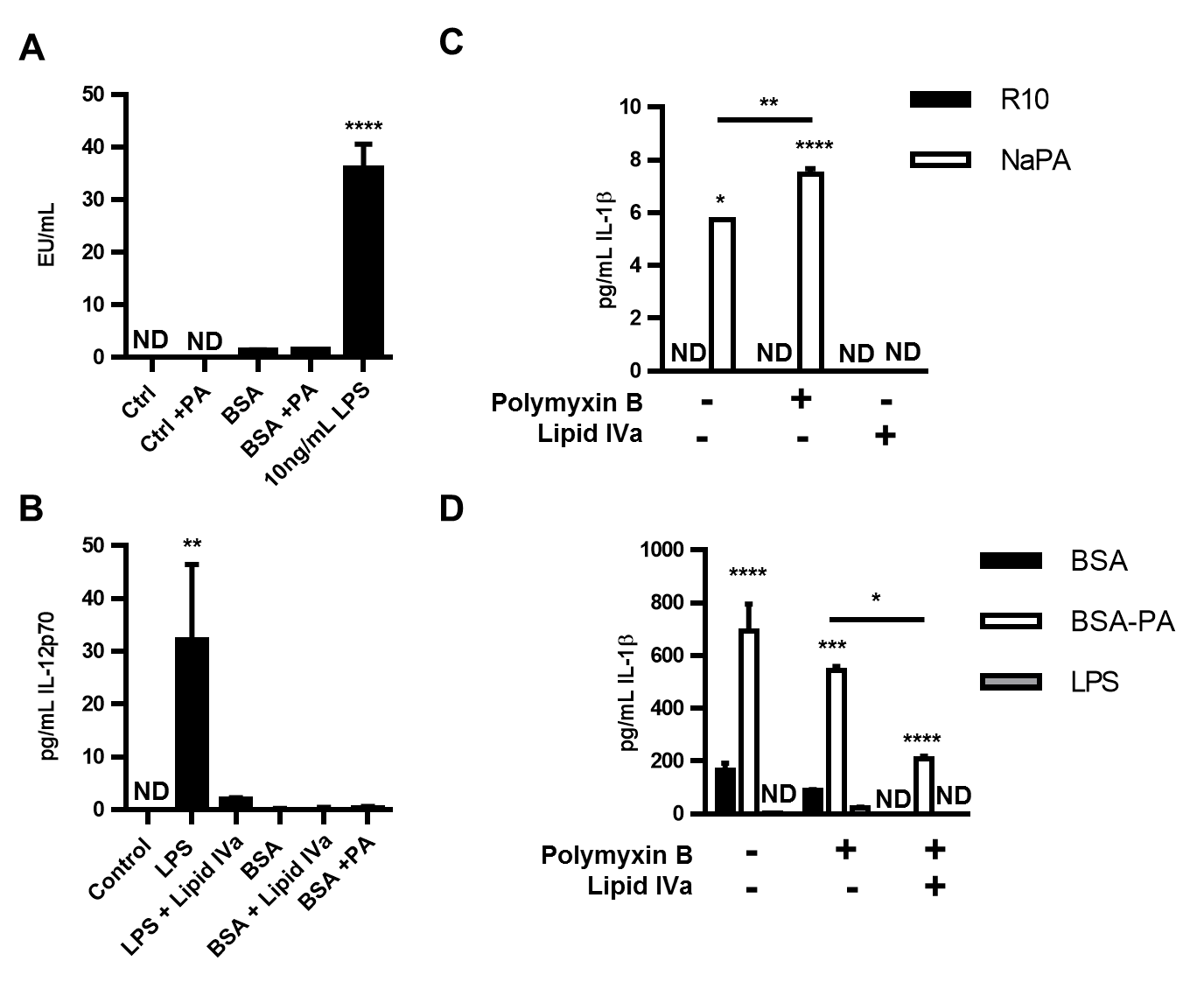

Supplement: S1 Fig — (A) Limulus assay of RPMI 1640 media with 10% FBS (Ctrl), Ctrl + 300μM PA, 150μM BSA, 150μM BSA + 300μM PA, and 10ng/mL LPS. One way ANOVA (** = p<0.01, *** = p<0.001, **** = p<0.0001). (B) MoDCs were treated with 150μM PA for 36hrs +/- Lipid IVa. The supernatant was analyzed for the concentration of IL-12. N = 3. One way ANOVA (** = p<0.01, *** = p<0.001, **** = p<0.0001). (C) MoDCs were treated with 150μM sodium palmitate (NaPA) +/- Polymyxin B or Lipid IVa for 36hrs. The supernatant was analyzed for the concentration of IL-1β. N = 4. (D) MoDCs were treated with 150μM palmitate solubilized with BSA (BSA-PA) +/- Polymyxin B or Lipid IVa for 36hrs. The supernatant was analyzed for the concentration of IL-1β. N = 4. ND = Not detectable. Two-way ANOVA (** = p<0.01, *** = p<0.001, **** = p<0.0001) compared to control unless indicated. All cytokines were detected using the Th17 multiplex kit (Millipore) and measured on the Bio-Plex® 3D System with Luminex xMap Technology (Bio-Rad), (more sensitive and with a larger dynamic range than the cytometric bead array system). (TIF) [file pone.0176793.s001.tif]

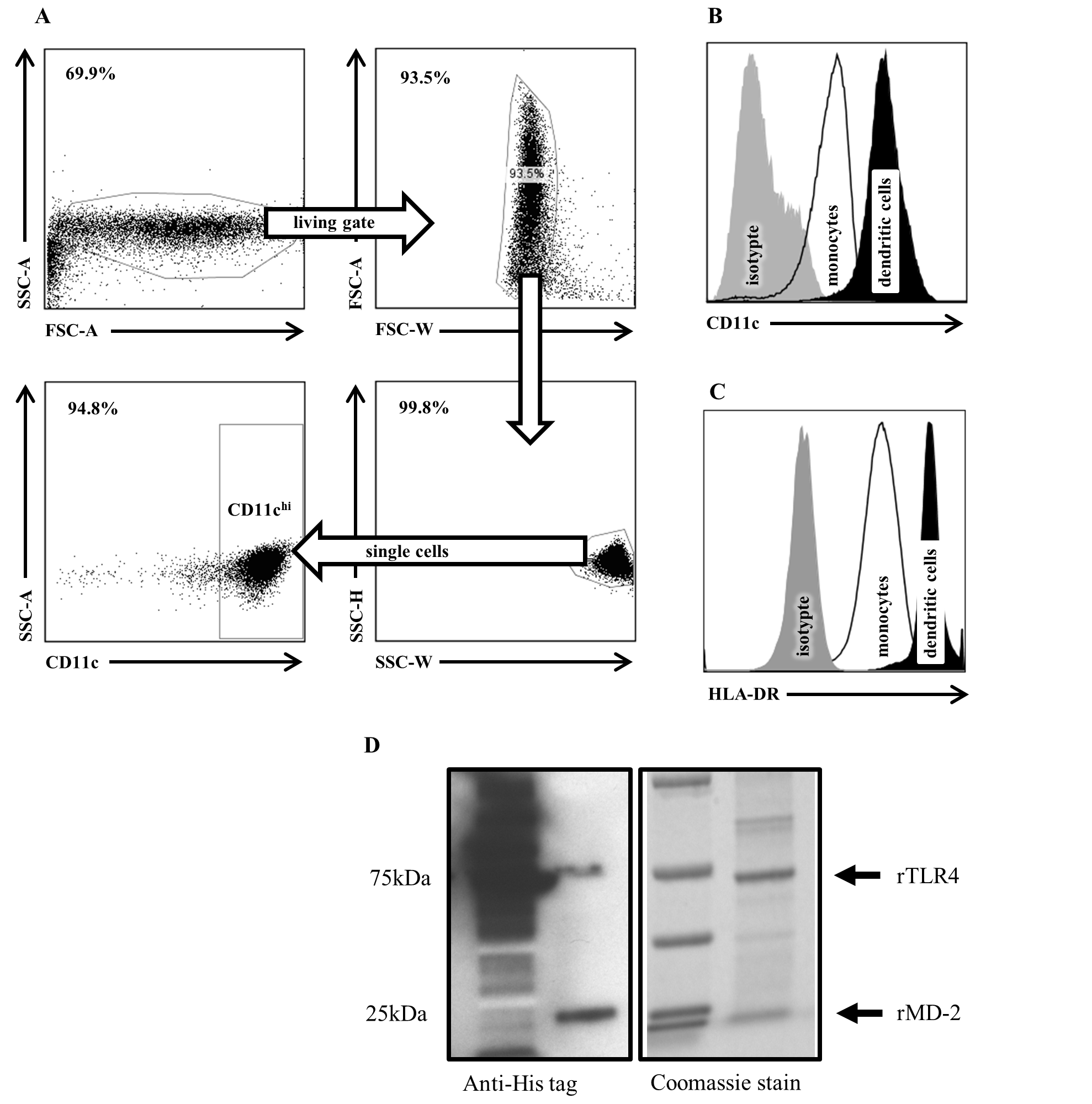

Supplement: S2 Fig — (A) MoDCs were gated first by FSC-A and SSC-A. Doublet discrimination was performed using FSC-H vs FSC-W and SSC-H vs SSC-W. Cells with the phenotype CD11chi were gated as MoDCs (>94%). (B) DCs express higher levels of CD11c than their precursor monocytes. (C) DCs express higher levels of HLA-DR than their precursor monocytes. (D) Western blot and Coomasie stain of SDS-PAGE of isolated rTLR4 and rMD-2 from Hela cells. (TIF) [file pone.0176793.s002.tif]

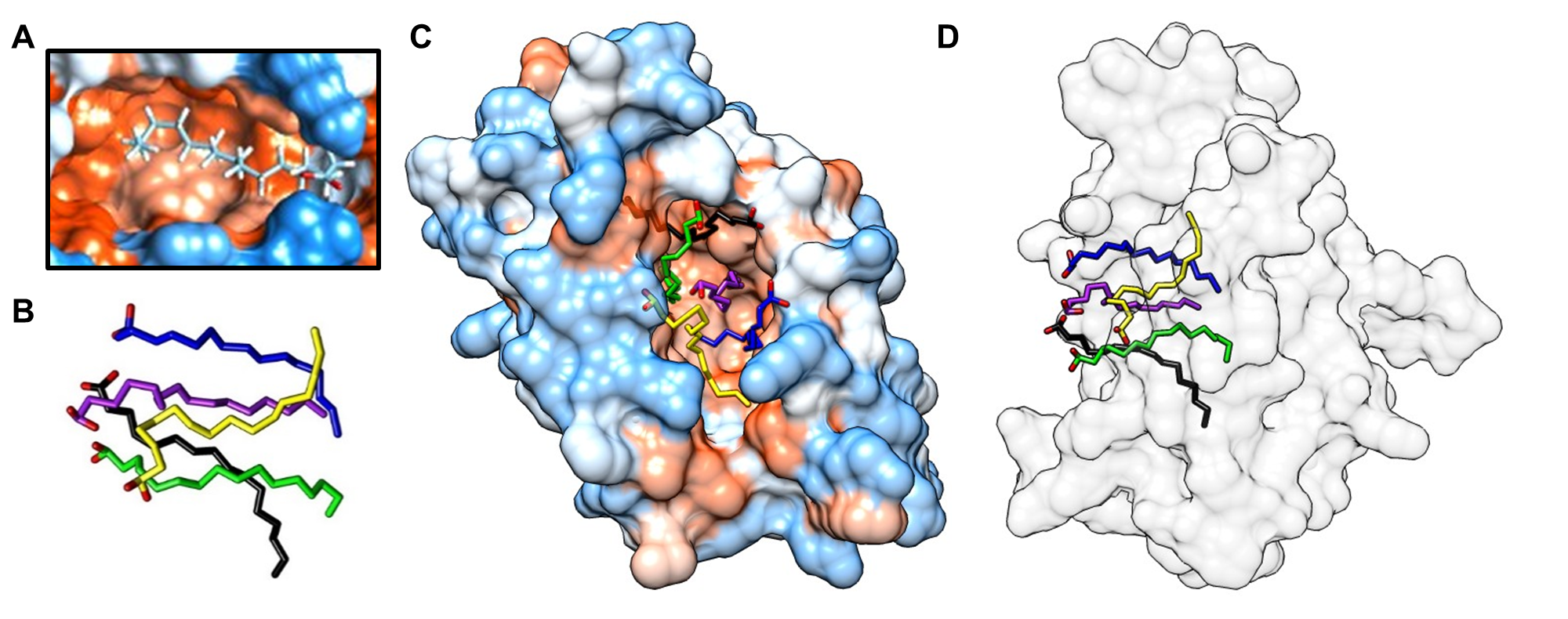

Supplement: S3 Fig — (A) SwissDock model of PA bound to the hydrophobic pocket of MD-2. Orange color indicates hydrophobicity of the protein and blue indicates hydropholicity. (B) Five palmitic acid molecules oriented based on the structure of LPS bound to MD-2. Each PA molecule is in a solid color (yellow, black, blue, green, and purple) with oxygen atoms in red. (C) Hydrophobicity molecular model of five palmitic acid molecules from (B) bound within the hydrophobic pocket of MD-2. (D) Model of five palmitic acid molecules bound to MD-2 (translucent). (TIF) [file pone.0176793.s003.tif]

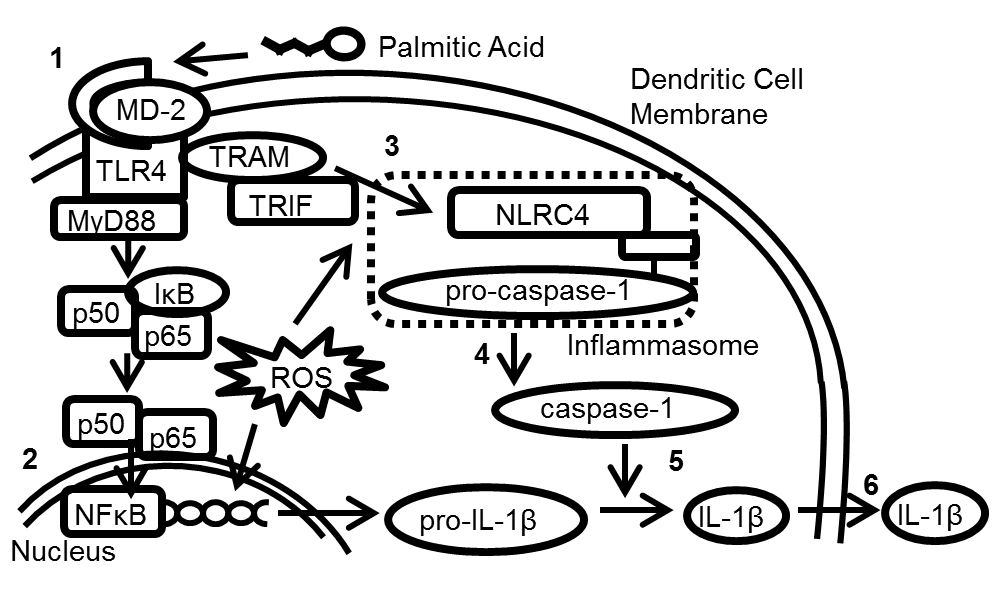

Supplement: S4 Fig — (1) PA binds TLR4/MD-2 and induces signal transduction resulting in NF-κB activation. (2) The canonical NF-κB signaling pathway induces transcription of the pro-IL-1-β gene which results in translation and protein expression. (3–4) PA-induced TLR4 signal transduction also results in activation of caspase-1, a process which occurs via inflammasome assembly. (5) Caspase-1 cleaves pro-IL-1-β into active IL-1-β. (6) With the secretory peptide signal exposed after cleavage, IL-1β is secreted from the cell. ROS has an undefined role in regulating this mechanism. (TIF) [file pone.0176793.s004.tif]

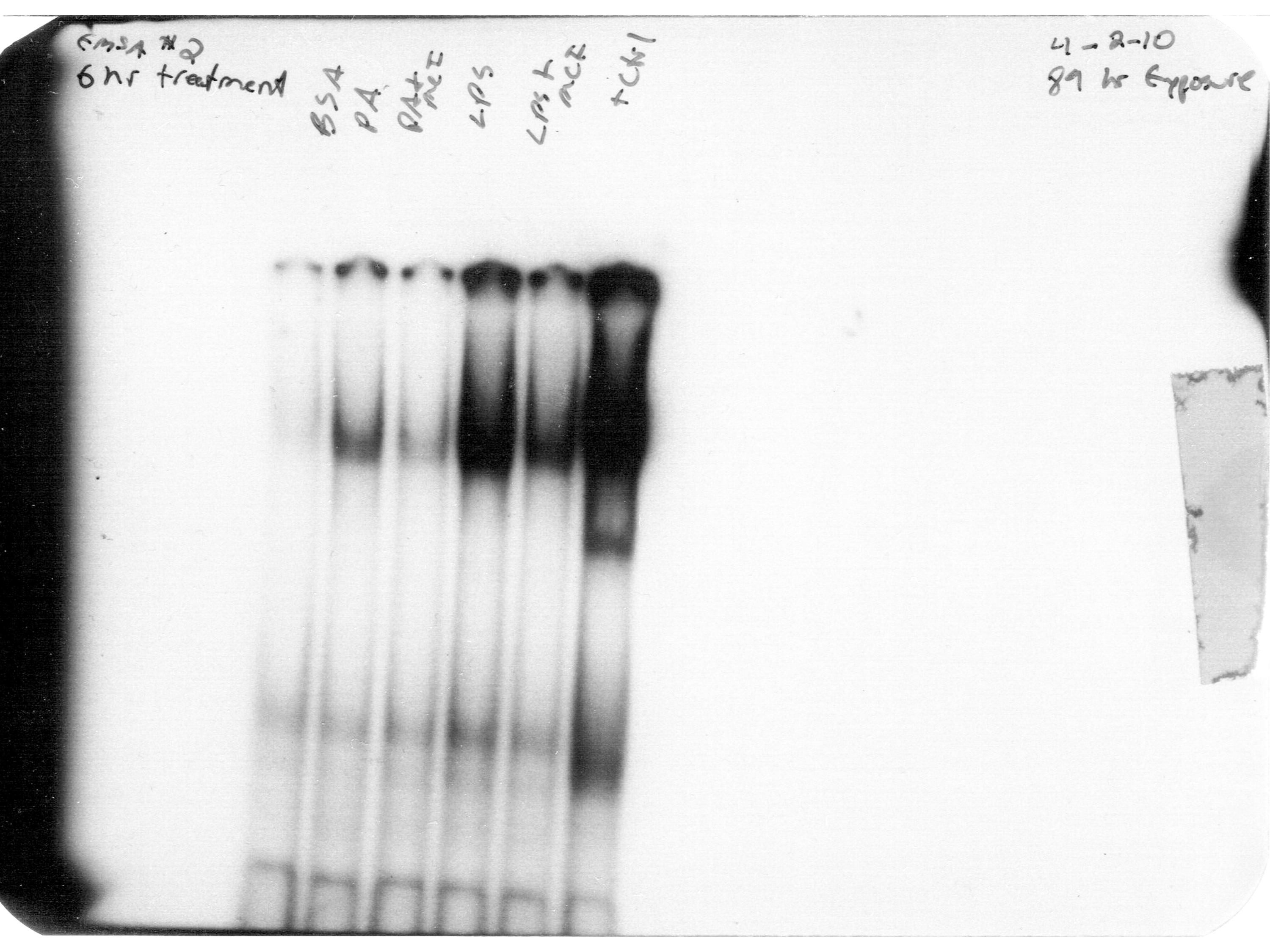

Supplement: S5 Fig — An uncropped image of Fig 6E. MoDCs were treated with 300μM PA+/- MCI-186 or LPS+/- MCI-186 for 3hrs and analyzed by EMSA. (TIF) [file pone.0176793.s005.tif]
